# Safety of Moxibustion: A Systematic Review of Case Reports

**DOI:** 10.1155/2014/783704

**Published:** 2014-05-26

**Authors:** Ji Xu, Hongyong Deng, Xueyong Shen

**Affiliations:** ^1^Shanghai University of Traditional Chinese Medicine, Shanghai 201203, China; ^2^Shanghai Research Center of Acupuncture & Meridian, Shanghai 201203, China

## Abstract

Moxibustion is a traditional medical treatment originating in China. It involves using the heat of burning moxa to stimulate acupoints. It is considered safe and effective and is widely used throughout the world. The increasing use of moxibustion has drawn attention to the procedure's adverse events (AEs). This review covers a total of 64 cases of AEs associated with moxibustion in 24 articles, reported in six countries. Some evidence of the risks of moxibustion has been found in these cases. AEs include allergies, burns, infection, coughing, nausea, vomiting, fetal distress, premature birth, basal cell carcinoma (BCC), ectropion, hyperpigmentation, and even death. The position, duration, distance between moxa and skin, proficiency of the practitioners, conditions of the patients, presence of smoke, and even the environment of treatment can affect the safety of moxibustion. Improving practitioner skill and regulating operations may reduce the incidence of adverse reactions and improve the security of moxibustion.

## 1. Introduction

Moxibustion is an ancient method of external therapy. It is based on the theories underlying traditional Chinese medicine (TCM). It usually involves stimulating acupoints with burning moxa wool. Moxibustion treatments can be classified as traditional moxibustion, drug moxibustion, and modern moxibustion. Traditional moxibustion is characterized by the use of moxa as a burning material and can be divided into direct moxibustion and indirect moxibustion depending on whether the moxa is in direct contact with the skin during the operation. In direct moxibustion, a moxa cone is placed directly on the skin and ignited. In indirect moxibustion, the moxa cone or stick is kept at a distance from the skin. The insulating materials used in indirect moxibustion can be air, garlic, ginger, aconite, salt, or other substances. In drug moxibustion, also called natural moxibustion, irritant drugs, such as cantharis, garlic, or semen sinapis (mustard seed), are coated on the surface of acupoints. This causes local skin to flush and blister, which is believed to alleviate disease. Modern moxibustion, such as microwave moxibustion, laser moxibustion, and electrothermal moxibustion, involves the simulation of traditional moxibustion stimulation factors through physical and chemical methods, which produces the therapeutic effects of moxibustion. The present review is mainly concerned with traditional moxibustion.

According to TCM theory, moxibustion has a dual effect, tonification and purgation. This involves the actions of the meridian system and the roles of moxa and fire. Studies have shown that the mechanism underlying moxibustion mainly involves the thermal effects, radiation effects, and pharmacological activity of moxa and the products of its combustion [1]. The effectiveness of moxibustion has been tested in the traditional and contemporary moxibustion clinic for more than 2500 years. A bibliometric analysis reported that up to 364 kinds of diseases can be treated with moxibustion. The most common indications of moxibustion therapy are malposition, diarrhea, and colitis. The next common indications are urinary incontinence and dysmenorrhea. The third common indications are knee osteoarthritis, temporomandibular joint disturbance syndrome, soft tissue injury, heel pain, asthma, urinary retention, and herpes zoster [2]. Moxibustion can also be used to treat weakness, fatigue, and problems related to aging [3].

The increasing use of moxibustion has drawn attention to its adverse events (AEs). Some case reports have described these adverse reactions, side effects, and complications of moxibustion. This review summarizes the published evidence regarding the safety of this medicinal approach.

## 2. Methods

### 2.1. Data Sources and Search Strategy

Four Chinese databases (SinoMed, CMCC, CNKI, and VIP) and three English databases (Medline, EMBASE, and Web of Science) were searched for case reports of AEs of moxibustion without any time or language restrictions. Search terms were “moxibustion, moxa, smoke,” combined with “safe, safety, adverse event, adverse reaction, side effect, complication, risk, and burn.” The literature searches were completed on November 20, 2013.

Review articles on safety of moxibustion were also searched. Two systematic reviews were retrieved, and the case reports cited in these reviews were examined too [4, 5].

### 2.2. Study Selection

Only firsthand case reports of complications or AEs of moxibustion are included in this review. Comments, letters, and investigations carried out on nonhuman were excluded. Some clinical trials of moxibustion that reported adverse events were excluded for reasons originally given by Lao et al., specifically that they were too small to provide convincing evidence of rare complications [6].

Two authors, Xu and Deng, carried out the screening and selection of articles independently. Disagreements were resolved through discussion.

### 2.3. Data Extraction

After screening, articles were read in full and extracted by two independent reviewers, Xu and Deng. Information extracted from each case included the author, year of publication, country of occurrence, number of patients affected, disease originally treated, preexisting conditions that might have contributed to the AEs, type of moxibustion, AEs reported and their outcomes, the practitioners' training, and the patients' status at follow-up. We also assessed the likelihood of causality between the events and moxibustion for each case. The causality assessment was based on the following five criteria [4]: time sequence of treatment and AEs, positional relationship between them, single risk factors, dechallenge (symptoms after cessation of moxibustion), and rechallenge (situations emerging during subsequent moxibustion). Each “yes” of the items was noted as 1 point. “No” and “not clear” were 0 points. The likelihood of each given AE was classified as certain (4-5), likely (3), possible (2), and unlikely (0-1) based on total scores. Disagreements in this procedure were resolved by discussion or by consultation with the third author, Shen.

## 3. Results

In this review, a total of 24 articles reported 64 cases of AEs associated with moxibustion (Table 1). They were from six countries, China, USA, South Korea, Spain, Japan, and Israel (Table 2).

The AEs of moxibustion reported in these articles include allergies, burns, infection, coughing, nausea and vomiting, fetal distress and premature birth, basal cell carcinoma (BCC), ectropion, hyperpigmentation, and other conditions (Table 3).

### 3.1. Allergy

There were seven cases of allergies associated with moxibustion. Six patients developed local skin flushing, swelling, and itching, and the last one reported a scattered red skin rash covering her whole body. Three patients recovered after 3–7 days of antiallergy treatments, and four recovered immediately after cessation of moxibustion treatment. All allergic reactions occurred in female patients and were reported in China (published in Chinese).

Zhang and Yang reported that a 36-year-old woman experienced mild allergies after moxibustion treatment [7]. This patient also had dysmenorrhea and was treated with acupuncture and indirect moxibustion on the lower abdomen. She underwent more than eight rounds of treatment and experienced redness and itching of the navel skin three times. The skin allergies disappeared the next day and never recurred in subsequent moxibustion treatments.

Feng and An reported a 66-year-old woman who received acupuncture and moxibustion treatments for her shoulder pain [8]. On the third day of moxibustion, she experienced rashes and itching on her neck and shoulders. The symptoms disappeared within 1 day of cessation of moxibustion, but they recurred when her roommates underwent moxibustion therapy in their shared apartment.

Hou reported a patient with a painful puerpera on her right wrist [9]. This was treated with acupuncture combined with moxibustion. On the second day of therapy, she experienced redness, itching, and rashes on her face, neck, and chest. The reactions disappeared if only the moxibustion was ceased. It recurred upon subsequent moxibustion.

Li and Liu reported that a 28-year-old woman received moxibustion after cupping and acupuncture for her facial paralysis [10]. Indirect moxibustion was performed with lighted moxa stick on the right Yifeng (SJ17) and the lower front of the earlobe. Two days after the treatment, the patient's right auricle became swollen and red without pain or itching. When the practitioner ceased moxibustion, the patient continued to undergo cupping and acupuncture, and the symptoms disappeared. When the moxibustion was applied nine days later, the patient's right cheek was swelling again.

Li and Liu reported two allergic reaction cases of moxibustion [11]. One patient's mother, 32 years old, accompanied her son to his moxibustion and waited with him in the room during his treatment. Three days later, she was admitted to a hospital for obvious swelling of her head and face. She was discharged after three days of antiallergy treatments. Another 22-year-old girl was cauterized at Shenque (RN8) with a moxa stick to reduce menstrual pain and experienced redness, itching, and papules of the navel. Her allergic symptoms and signs disappeared one week later.

Wang reported an allergic reaction caused by excessive inhalation of moxa smoke [12]. A 28-year-old woman underwent two rounds of moxibustion in an open treatment room without any adverse reactions. However, during the third round, she underwent moxibustion in a closed room in which she later slept. When she woke up, she experienced chest tightness, suffocation, difficulty breathing, throat discomfort, irritability, itching, and scattered papules. The allergies disappeared after three days of antiallergy treatments. Seven days later, similar adverse reactions recurred and again resolved. They never reappeared after the moxibustion had ceased.

### 3.2. Burns

There were 43 cases involving burns. These were reported in 6 articles. Most of the burns were in newborns, children, and individuals with sensory disabilities. The symptoms of burns caused by moxibustion included pain, local skin blisters, ulcers, and secondary infections. They were usually curable with symptomatic treatments. Another large problem with the more severe burns was permanent and disfiguring scarring. These were associated with direct moxibustion.

Li reported an 18-year-old girl diagnosed with right lower limb paralysis [13]. Treated with indirect moxibustion after acupuncture, the patient's right Yanglingquan (GB34) suffered minor burns and then blistering and ulceration. After 40 days of symptomatic treatment, the wound finally healed. In this case, the patient had sensory disturbance of the right lower limb and could not tell the practitioner administering the moxibustion that she was feeling any pain, which increased the risk of burns.

Li reported 37 cases of newborn burns and infections associated with improper moxibustion from 1990 to 1997 [14]. These newborns had been treated with spark moxibustion, a folk form of moxibustion used to treat neonatal jaundice, most commonly by means of a burning object such as a rush tapped quickly on the skin. All patients showed varying degrees of burns, 34 mild and 3 moderate. Eight patients were diagnosed with secondary systemic infections, including one case of staphylococcal scalded skin syndrome (SSSS), six cases of septicemia, and one case of pyemia. The author inferred that improper and excess moxibustion were the main causes of these burns and infections.

Condé-Salazar et al. reported a 40-year-old male with numerous burns on the wrist and ankle [15]. The patient, who lived in Spain, received acupuncture and moxibustion therapy for his tennis elbow and experienced these burns as a result. However, the moxibustion therapy did cure his complaints effectively.

Reinhart and Ruhs reported an Asian boy, two and a half years old, who had numerous punched-out, circular burns distributed on his face, trunk, and extremities [16]. They were remarkably symmetrical in most areas. The boy had sustained the skin lesions when his father performed folk moxibustion after a visit to an American physician had failed to cure his child's illness. In this case, the patient's father was not an actual practitioner; he was attempting to repeat the practice as recalled from his childhood.

Carron et al. reported two cases of third-degree burns associated with moxibustion [17]. A 58-year-old white man suffered a long history of cervical pain and spasm aggravated by a postural deformity resulting from painful plantar calcaneal spurs. When conventional therapy failed to relieve his symptoms, the patient went to a local acupuncture clinic for acupuncture, scraping, and moxibustion treatments. Examination after more than three rounds of therapy showed two painful, third-degree burns, 3 mm in diameter, 3 mm deep, covered with black eschar and surrounded by a 3 × 4 cm area of erythema on the left heel. Another 62-year-old man had undergone three lumbar laminectomies and a dorsal rhizotomy for relief of pain of sciatic distribution. He sought acupuncture therapy at a clinic and was instructed to return home to continue his moxibustion therapy. Six months later, when the patient again sought medical attention, permanent scars were present at all moxibustion sites.

Fisman reported a 38-year-old Cambodian man presenting symmetrical hyperpigmented maculae on the abdomen [18]. These unusual skin findings had been caused by burns from the application of small pieces of smoldering cloth.

### 3.3. Infection

There were six cases of infection involving moxibustion. They were reported in Korea, the U.S., and Israel. Of these cases, three were surface tissues infections, two were deep organ infections, and one was hepatitis C virus (HCV) infection.

Chau reported a 53-year-old Korean woman who was admitted to a hospital with a diagnosis of cellulitis [19]. Before that, the patient, though untrained in Chinese medicine, had attempted to self-administer direct moxibustion for intermittent headaches and experienced cellulitis on her instep as a complication. She was treated with intravenous antibiotics for 24 hours and discharged in a good condition.

Chong et al. reported a* Pasteurella multocida* infection of the calf associated with moxibustion [20]. A 48-year-old woman from South Korea had a history of prednisolone treatment combined with acupuncture and moxibustion for her degenerative arthritis.* P. multocida* infection is rare in humans, but this organism was isolated and identified from fluid aspirated from this woman's calf. It was probable that the organism entered through the burn wound ulcers.

Choi et al. reported a 69-year-old man with pyogenic liver abscess (PLA) following acupuncture and moxibustion treatment [21]. This patient had received acupuncture on his arms and moxibustion on his abdomen three times per week for insomnia. About one month later, he felt nauseous and feverish and lost about 9 kg of body weight. An abdominal computed tomography (CT) scan with contrast revealed multiseptated cystic lesions in the right and left lobes of liver, the largest, which was in the left lobe, measuring about 10.0 cm. Pyogenic abscess was confirmed by ultrasound-guided percutaneous needle aspiration with Gram stain and culture of the aspirate. In this case, authors assumed that the patient had* Streptococcus intermedius* bacteremia after being treated with contaminated acupuncture needles and* Streptococcus intermedius* may have been seeded in the liver. The time sequence of the symptoms and the treatments suggested a possible causal relationship, but there was no direct evidence of that.

Lee et al. reported a spinal epidural abscess originating from cellulitis after moxibustion [22]. A 78-year-old woman was suffering from diabetes mellitus, hypertension, lower back pain, and knee osteoarthritis. She had cauterized her right third finger with moxa repeatedly over 4 years and had experienced recurrent infections with intermittent pus discharge. The authors believed that the patient's spinal epidural abscess originated from osteomyelitis and cellulitis of the finger secondary to burn injuries caused by repeated moxibustion.

Bardia et al. reported a 66-year-old Somalian man diagnosed with hepatocellular carcinoma secondary to cirrhosis due to hepatitis C [23]. The patient did not have the usual risk factors for hepatitis C but had undergone moxibustion and cutting of the wrists and abdomen using sharp objects or needles to release the bad blood. It is possible that he acquired hepatitis C through the sharing of infected knives or the burn wound associated with his moxibustion therapy.

Sternfeld et al. reported one case of* Serratia marcescens* infected silk suture rejected by combined acupuncture, moxibustion, and low-power laser therapy from the abdominal fascia [24]. An 82-year-old Israeli woman was complaining of right hypochondrial pains caused by a forgotten foreign body that had been implanted in her body during a cholecystectomy surgery twelve years earlier. Both acupuncture and moxibustion supplemented by low-power laser beam were performed on the site of infection. Acupuncture and low-power laser beam treatment were carried out by doctors at the clinic; patient was self-treated by moxibustion. After 20 sessions, a silk surgical suture was expelled. From then on recovery was spontaneous and fast.

### 3.4. Fetal Harm

There were two cases of fetal harm caused by moxibustion reported in China.

Cai reported one case of fetal death following moxibustion [25]. A 26-year-old woman was diagnosed with breech presentation during the eighth month of pregnancy and was told to treat herself using moxibustion at Zhiyin (BL67) to correct it. About ten minutes into her third treatment, the pregnant woman felt chest tightness, palpitations, dizziness, vomiting, and increased fetal movement. She was admitted to hospital with a diagnosis of umbilical cord around the neck of the fetus and fetal distress. There was no improvement after oxygen and drug therapy, and the fetus died three hours after her admission.

Su reported a case of premature delivery caused by moxibustion [26]. A pregnant woman suffered breech presentation at 29-week gestation and was treated with moxibustion combined with chest-knee position therapy. Seven hours after the treatment, the patient felt lower abdominal pain continuously. This pain grew progressively more intense. Three hours later, a large amount of clear liquid was discharged from her vagina, followed by an object which became lodged in the vagina. Emergency examination showed that the fetus had been delivered incompletely in breech presentation and had already died due to suffocation.

### 3.5. Other Adverse Events

Some rare adverse events related to moxibustion, such as coughing, nausea and vomiting, basal cell carcinoma, ectropion, xerosis, and death, were also recorded.

Wen et al. reported three cases of AEs associated with moxibustion in China [27]. A 47-year-old woman was admitted for chemotherapy after colorectal cancer surgery and sought moxibustion to reduce the discomfort induced by drugs. She was treated with moxa stick at the points of Zhongwan (RN12), Danzhong (RN17), Qihai (RN6), and Guanyuan (RN4). At 15 minutes into the first therapy, the patient felt nausea and vomiting, which recurred during the next day's moxibustion. A 51-year-old man presented similar symptoms. The third patient, a 68-year-old woman, was admitted to the hospital for her lung cancer complicated by pulmonary infection. She complained of the smell of moxa smoke and coughing when her roommates carried out moxibustion.

Yun et al. reported a large superficial basal cell carcinoma (BCC) arising from a burn scar secondary to repeated moxibustion [28]. A 58-year-old Korean man had applied moxa cautery to the same site repeatedly for relief of abdominal pain over ten years. He presented with a 3-year history of a dark reddish plaque on the lower part of the abdomen. Physical examination revealed a well-demarcated, round, 7.2 × 5.7 cm dark reddish plaque with some brown and black crusts and pigmentation on the lower part of the abdomen. Histopathological examination showed nests of basaloid cells arising from basal layers of the epidermis and extending into the dermis. There was peripheral palisading of the nuclei of the tumor cell nests and peritumoral lacunae between the tumor cells and stroma. Diagnosed as BCC, the lesion responded well to radiation therapy and there was no evidence of recurrence 5 years later.

Chen reported a case of a loose lower eyelid and ectropion after moxibustion on the craniofacial points [29]. A 41-year-old Chinese woman suffered from the left facial paralysis. She was treated with direct moxibustion at Dicang (ST4), Jiache (ST6), Yangbai (GB14), and Sibai (ST2). After therapy lasting a few days, the patient was better but developed an eversion and sagging of the left lower eyelid. The author believed it to be a consequence of improper moxibustion at Sibai (ST2), where the infraorbital nerve, zygomatic branch of the facial nerve, and infraorbital artery are distributed.

Ogata et al. reported a 29-year-old Japanese man with bronchial asthma who died while undergoing acupuncture and moxibustion treatment [30]. The patient had suffered from periodic asthma attacks for nine years, and the asthmatic attacks became more frequent and persisted over longer periods during the last year before his death. After being treated with three rounds of moxa followed by two needles inserted for acupuncture, he suddenly collapsed and died. The autopsy findings of the lungs in this case were compatible with a diagnosis of severe asthma. The tissue injuries to the patient from the needles and the burns from the moxa treatment were found to be mild. The apparent cause of this death was respiratory dysfunction due to a severe asthmatic attack. It can be speculated that his death from asthma might have been associated with emotional stress and apprehension that he experienced while undergoing acupuncture and moxibustion for the first time.

## 4. Discussion

### 4.1. Traditional Understanding of Moxibustion Safety

Ancient literature on Chinese medicine rarely reported AEs of moxibustion, but they did indicate two issues regarding moxibustion safety. First, there are the contraindications of moxibustion, which are based on the TCM theory. For example,* Treatise on Febrile Diseases* advises the reader, “Be cautious and do not apply moxibustion when the patient's pulse is weak and fast.” It is also suggested that some points that are located close to important organs, nerves, and blood vessels or where local subcutaneous tissue is thin be avoided during moxibustion to prevent injuries. In the* Zhen Jiu Jia Yi Jing*, moxibustion is forbidden on 28 acupoints. The ancients believed that acupoints on the head, face, and the distal ends of the limbs should not be used or used sparingly to reduce the intensity of moxibustion.

### 4.2. Experimental Studies on the Temperature and the Products of Moxibustion

Concerns about safety of moxibustion have only been raised in recent decades. There are two approaches to evaluate the safety of moxibustion: experimentation and observation in clinical settings. The therapeutic effects of moxibustion are generally believed to come mainly from thermal effects, radiation effects, and the pharmacological actions of the products of moxa combustion. Studies of the safety of the radiation used in moxibustion are rare, and the focus has been placed mostly on the other two factors, thermal effects and products of combustion. Burning moxa without flame can produce temperatures of about 548–890°C [31, 32]. Some people think that this treatment is essentially a physical thermal effect [33]. Other experiments have confirmed that a single Zhuang (unit of dosage of moxibustion) of moxa cone (2 mg) moxibustion administered to the mouse abdomen can raise the temperature of the mouse's skin to 130°C and that of the area beneath the skin to 56°C [34]. Some have observed the impact of moxibustion at different distances on local skin temperature. The safe distance for indirect moxibustion seems to be 3-4 cm [35, 36]. The temperature of burning moxa can play a therapeutic role, but it offers a risk of burns if administered improperly.

Many of the components of the moxa and the products of their combustion have been identified [37, 38]. They include a variety of biological activities and play important roles in the comprehensive effects of moxibustion [39–41]. Some products of moxa combustion are brown and tar-like. They take effect after entering the human body through burn-damaged skin. Another product of moxa combustion is smoke. Moxa smoke contains many complex components, and its volatile ingredients include ammonia, alcohols (ethylene glycol, pentyl-butanol), aliphatic hydrocarbons, aromatic hydrocarbons, terpene compounds, and their oxides. They may come from products of incomplete combustion, volatile oil, and the products of the oxidation of that oil. There is debate regarding the security of moxa smoke. The mugwort leaf contains terpenes. It may produce polycyclic aromatic carcinogens during combustion. During moxibustion, the concentration of air pollutants, such as nitrogen oxides, carbon monoxide, and particulates, is ten times higher than standard class II as issued by the State Environmental Protection Act. These substances can damage the health of patients and staff [42]. Acute toxicological testing of moxa smoke on SPF degree SD rats showed the median lethal concentration (LC_50_) to be 11,117 mg/m^3^. It was therefore classified as minimally toxic according to WHO standards [43]. An AMES test showed moxa smoke condensation (MSC) with metabolic activation using a liver fraction (S-9) to enhance the mutagenic activity of* Salmonella* strain TA98. This means that MSC may be mutagenic [44]. However, one study gave consideration to short-term and long-term exposure. It showed that the volatile matter and carbon monoxide generated by moxa smoke under normal operating conditions did not exceed safety levels [45].

### 4.3. Analysis of the AEs in Moxibustion Included in This Review

Many large-sample surveys of the safety of acupuncture have been carried out around the world [46–48]. More than 762 cases (in 219 articles) of AEs related to acupuncture were reported before 2011 [49]. There are far fewer clinical safety survey data for moxibustion and far fewer case reports of AEs. Two previous reviews have been conducted on the safety of moxibustion treatment. Parka reviewed 13 case reports (7 clinical trials and 1 prospective survey) of AEs related to moxibustion [4]. Xu et al. reviewed 4 (294 acupuncture and 10 cupping) [5]. The present review analyzed 64 cases in 24 articles, covering all cases cited in the two articles above. These cases were mainly found in China (51 cases/12 articles), the U.S. (5/6), and Korea (4/4). This is consistent with the fact that there is far more use of moxibustion in these areas than elsewhere in the world. Adverse reactions do not seem to be related to patient age (from newborn to 82 years old). The most common risk of moxibustion was found to be burns (43 cases/6 articles), which are caused by the high combustion temperature of moxa. Burns can be caused by direct contact with the products of combustion (direct moxibustion) or by radiant heat (indirect moxibustion). However, the issue of whether moxibustion-induced burns are really an adverse event must be addressed. In traditional moxibustion, also known as scarring moxibustion or suppurative moxibustion, local minor burns, purulence, and scarring during treatment were always considered normal. Most ancient Chinese doctors were in favor of using scarring moxibustion. They believed “where there is moxibustion scar, there is cure.” Scarring moxibustion is still used in modern clinical practice. Although accidental burns may happen during moxibustion, it was here noted that some patients in reports of burns accepted these skin lesions as the natural results of the therapy. For this reason, to determine whether a burn is a side effect of moxibustion or not, the doctors' expectations and patients' acceptances should be taken into account. The final therapeutic effects may have a nonignorable influence on this assessment.

Allergies (7 cases/6 articles) and infections (6 cases/6 articles) were reported at frequencies similar to those of other adverse reactions to moxibustion. However, the causality between allergies and moxibustion was stronger (average likelihood score: 3.29) than that between the infections and the moxibustion (average likelihood score: 1.83). Most allergic reactions occurred at the local level and may have been caused by physical (e.g., heat) and chemical (e.g., tars, ammonia, and alcohols) stimulations associated with moxa combustion. Only one case of full-body allergy was reported. It was due to excessive inhalation of smoke in a closed room. A survey of moxibustion practitioners in five Chinese medicine hospitals in Guangdong province showed that moxibustion smoke raised the incidence of chronic laryngitis from 3.70% (nonacupuncturists) to 26.67% (acupuncturists) (*P* < 0.05) [50]. Local skin infections were usually secondary to burns. Deep organs infections (e.g., liver abscess, spinal epidural abscess) and viral infection (e.g., hepatitis C) could be secondary to local infections. Very weak causality was found between nonlocal infections and moxibustion.

Moxibustion at Zhiyin (BL67) is often used to correct abnormal presentation in TCM. Recently, a multicenter randomized controlled trial showed moxibustion at acupoint BL67 to be an effective and safe means of correcting nonvertex presentation when used between 33 and 35 weeks of gestation [51]. However, the two cases of fetal distress and premature delivery presented here indicate the potential risks of fetal harm related to moxibustion. Nausea and vomiting after moxibustion were observed solely in cancer patients who were undergoing chemotherapy and were sensitive to the smell, which can easily cause gastrointestinal reactions. The case of loose lower eyelids and ectropion after moxibustion indicated the potential risk of damage to superficial nerves and vessels. In the case of BCC secondary to long-term moxibustion, it is possible that the burn scar secondary to moxa can be repeatedly cauterized. This caused the development of BCC in an area protected from sun exposure. It is rare for reports of death after moxibustion. In the only death evaluated here, the causality between the consequence and moxibustion was assessed as unlikely, but the effects of stress and other consequences of moxibustion should not be ignored, especially considering that this patient was entirely new to moxibustion.

## 5. Conclusion

Moxibustion is considered a safe and effective traditional therapy, and large numbers of clinical reports have indicated that it is effective and associated with few adverse events. There is no absolutely safe treatment in the world, and moxibustion may be also related with some security risks in certain circumstances. Although the studies examined here included some mistakes, such as nonaccidental injuries as side effects, and some adverse events have little causal relationships with moxibustion, the evidence collected here was consistent with that of previous reports indicating the risks of moxibustion. The position, duration, distance between moxa and skin, proficiency of doctors, patient conditions, stimulations from smoke, and even the environment of treatment can affect the safety of moxibustion. The exact causes of most of these AEs cannot be determined. This issue should be addressed in further experimental studies, clinical trials, and case reports on the safety of moxibustion. Improving practitioner skills, regulating the operations, and controlling for time, distance, dose, and protection can reduce the incidence of adverse reactions and improve the safety of moxibustion.

## Figures and Tables

**Table 1:** Case reports of AEs associated with moxibustion.

Adverse events	Number of reports	Number of cases
Allergies	6	7
Burns	6	43
Infection	6	6
Nausea or vomiting	1	2
Cough	—	1
Fetal harm	2	2
Basal cell carcinoma	1	1
Ectropion	1	1
Death	1	1

Total	24	64

**Table 2:** Countries in which AEs were reported to be associated with moxibustion.

Country	Number of reports	Number of cases
China	12	51
U.S.	5	6
Korea	4	4
Spain	1	1
Japan	1	1
Israel	1	1

**Table 3:** Adverse events associated with moxibustion.

Author/year/country	Cases age/sex	Disease treated	Moxibustion treatment	Adverse event	Practitioner	Follow-up	Causality
Zhang and Yang 2006, China	36 (F)	Dysmenorrhea	Indirect moxibustion with moxa box	Local allergy	Practitioner	Recovered (1 d)	Possible (1, 1, 0, 0, 0)
Feng and An 2005, China	66 (F)	Shoulder pain	NC^a^	Local allergy	Practitioner	Recovered (1 d)	Certain (1, 1, 0, 1, 1)
Hou 2008, China	1 (F)	Right wrist pain	NC	Local allergy	Practitioner	Recovered	Certain (1, 1, 0, 1, 1)
Li and Liu 2000, China	28 (F)	Facial paralysis	Indirect moxibustion with moxa stick	Local allergy	Practitioner	Recovered	Certain (1, 1, 0, 1, 1)
Li and Liu 2008, China	32 (F)	NC	Stay in moxibustion room	Local allergy	NC	Recovered (3 d)	Possible (1, 0, 1, 0, 0)
	22 (F)	Menstrual abdominal pain	Indirect moxibustion with moxa stick	Local allergy	Practitioner	Recovered (7 d)	Certain (1, 1, 1, 1, 0)
Wang 1998, China	28 (F)	Dyspepsia	Moxibustion in a closed room	Full-body allergy	Self	Recovered (3 d)	Likely (1, 0, 1, 0, 1)
Li 2002, China	Newborns	NC	Spark moxibustion	Burn and infection	NC	NC	Certain^b^ (1, 1, 1, 0, 0)
Li 1989, China	18 (F)	Lower limb paralysis	Moxibustion with moxa stick	Burn	Practitioner	Recovered (40 d)	Certain (1, 1, 1, 0, 0)
Condé-Salazar et al. 1991, Spain	40 (M)	Tennis elbow	NC	Burn	Practitioner	NC	Certain (1, 1, 1, 0, 0)
Reinhart and Ruhs 1985, U.S.	2.5 (M)	Hyperthermia and seizures	Folk moxibustion	Numerous burns	Patient's father	Recovered (1 y)	Certain (1, 1, 1, 0, 0)
Fisman 2002, USA	38 (M)	Hepatic cirrhosis	NC	Lesion after burn	NC	NC	Certain (1, 1, 1, 0, 0)
Carron et al. 1974, U.S.	58 (M)	Cervical pain and spasm	Direct moxibustion	Burn	Practitioner	NC	Certain (1, 1, 1, 0, 0)
	62 (M)	Pain	NC	Burn	Self	NC	Certain (1, 1, 1, 0, 0)
Chau 2006, U.S.	53 (F)	Intermittent headaches	Scarring direct moxibustion	Cellulitis	Self	Recovered	Likely (1, 1, 1, 0, 0)
Chong et al. 1982, Korea	48 (F)	Arthritis	Moxa cauterization	*Pasteurella multocida *infection of the calf	NC	NC	Possible (1, 1, 0, 0, 0)
Choi et al. 2013, Korea	69 (M)	Insomnia	Jang-chim moxibustion	Pyogenic liver abscess	Practitioner	Recovered (22 d)	Unlikely (0, 1, 0, 0, 0)
Lee et al. 2008, Korea	78 (F)	Diabetes mellitus	Repeated moxibustion over 4 years	Spinal epidural abscess	Self	Recovered (170 d)	Possible (1, 0, 1, 0, 0)
Bardia et al. 2006, USA	66 (M)	NC	Moxibustion and cutting	Hepatitis C	Practitioner	NC	Unlikely (1, 0, 0, 0, 0)
Sternfeld et al. 1988, Israel	82 (F)	Hypochondrial pain	Indirect moxibustion combined with acupuncture and low-power laser exposure	Infection	Self	Recovered	Possible (1, 1, 0, 0, 0)
Cai 1999, China	26 (F)	Breech presentation	Moxibustion at BL67	Fetal death	Self	Fetal death	Possible (1, 0, 1, 0, 0)
Su 1999, China	1 (F)	Breech presentation	Moxibustion combined with chest-knee position therapy	Premature delivery	Self	Fetal death	Unlikely (1, 0, 0, 0, 0)
Wen et al. 2011, China	47 (F)	Chemotherapy after colorectal cancer surgery	Moxibustion with moxa stick	Nausea and vomiting	Practitioner	Recovered	Likely (1, 0, 1, 0, 1)
	51 (M)	Chemotherapy after lung cancer surgery	Moxibustion with moxa stick	Vomiting and insomnia	Practitioner	Recovered	Possible (1, 0, 1, 0, 0)
	68 (F)	Lung cancer complicated with pulmonary infection	Inhalation of moxa smoke	Cough	NC	Recovered	Likely (1, 0, 1, 1, 0)
Chen 1998, China	41 (F)	Facial paralysis	Direct moxibustion	Lower eyelid loose and ectropion	Practitioner	NC	Likely (1, 1, 1, 0, 0)
Yun et al. 2009, Korea	58 (M)	Abdominal pain	Moxibustion on the same site repeatedly 10 years	Basal cell carcinoma	NC	No recurrence (5 y)	Likely (1, 1, 1, 0, 0)
Ogata et al. 1992, Japan	29 (M)	Asthma	Moxibustion and acupuncture	Death	Practitioner	Death	Unlikely (1, 0, 0, 0, 0)

^a^"NC": not clear.

^b^Although the scores for these burn cases were less than 4, they were classified as “certain” because they had been directly observed to have been caused by moxibustion.
